# Linking Disaster Predictions to Health Care Strain and Costs: A Novel Military-Civilian Case Study

**DOI:** 10.1017/dmp.2026.10338

**Published:** 2026-03-30

**Authors:** Sarah McCuskee, Kevin Petrozzo, David G. Buckler, Ellerie Weber, Yosef Travis, Lauren M. Sauer, Alexis Zebrowski

**Affiliations:** 1 Department of Emergency Medicine, Icahn School of Medicine at Mount Sinai, New York, NY, USA; 2 Mount Sinai Health System, New York, NY, USA; 3 Department of Population Health Science and Policy, Icahn School of Medicine at Mount Sinai, New York, NY, USA; 4 Department of Environmental, Agricultural and Occupational Health, College of Public Health at University of Nebraska Medical Center, Omaha, Nebraska, USA

**Keywords:** Disaster planning, diagnosis-related groups, *International Classification of Diseases*, military medicine

## Abstract

**Objective:**

Broad predictions about disaster health care needs are insufficiently granular to estimate system impacts. Historical utilization data could refine predictions, but disaster patients differ systematically from usual health care. This study matches civilian health care utilization data to predicted disaster patient characteristics and validates the method, using the theoretical example of mass military patient transfer to civilian hospitals.

**Method:**

An ICD-10 code sorting algorithm was developed, categorizing each ICD-10 code into one of 13 broad stakeholder-predicted categories. Blinded clinicians validated each categorization. Healthcare Cost and Utilization Project (HCUP) and civilian hospital billing data were used to match category/ICD-10 code pairs to Diagnosis-Related Groups (DRG) to understand utilization for each disaster injury category.

**Results:**

Agreement was excellent (Cohen’s ĸ = 0.86; 99.2% agreement among ≥2/3 clinicians). The resulting ICD-10 codes—disaster injury category crosswalk was applied to 1,945,272 HCUP inpatient encounters. Most disaster injury categories corresponded exactly to one DRG; some DRGs, e.g., multi-system trauma, corresponded to multiple disaster injury categories. Length of stay and payer varied by disaster injury category and HCUP vs hospital billing data.

**Conclusions:**

This method refines broad predictions about disaster epidemiology using linkage to granular civilian health care data; it can improve readiness by accurately modeling disaster care and reimbursement.

## Introduction

Health care readiness requires complex coordination between medical, surgical, public health, military, and financial resources.^1^ During disasters such as pandemics, conflicts, or environmental catastrophes, surges in patient demand strain hospital systems’ operational capabilities and existentially threaten health system financial stability.^2^^–^^4^ To prepare for such events and ensure financial and operational sustainability, civilian hospital systems can simulate and plan for different disaster scenarios, and can leverage historical utilization and discharge data to define the characteristics of expected patients.^5^ However, such simulations are often challenging because characteristics of patients and care provided during disasters differ systematically from those in usual health care operations.^3^ In this paper, we develop a method to connect broad predictions about medical characteristics of a specific disaster population—namely injured or ill servicemembers transferred during an international large-scale combat operation (LSCO) to domestic care sites—with retrospective health care utilization data from a civilian population.

Generally, using retrospective data to estimate the costs and financial impact of novel disasters may be misleading. Broad predictions about the characteristics of disaster patients, such as those used in disaster planning,^6^^–^^8^ have unclear correlates in granular real-world health care utilization data.^8^ Additionally, taxonomies used for reimbursement, such as Diagnosis-Related Groups (DRGs) in the United States, are developed for usual health care operations and do not easily apply to disaster-related health care provision.^8^ Health care readiness and emergency planning thus must incorporate informed predictions about the disaster population with existing data derived from non-disaster populations.

This paper presents a flexible, informative algorithm for using historical data derived from real health systems to predict their individual financial and operational needs in various types of predicted disasters. The algorithm aims to increase the precision and realism with which both public health and health care systems plan for the operational and financial impacts of a disaster. To demonstrate ideas, we then apply the algorithm to a theoretical mass casualty transfer effort due to an international military LSCO. In the theoretical scenario, injured and ill servicemembers and civilians are transferred to receive health care in civilian health care facilities in the Omaha, Nebraska, USA, area through the National Disaster Medical System (NDMS).

Cross-sectoral readiness strategies such as the military-civilian cooperation used in the theoretical disaster example require flexible data harmonization for planning purposes, such as that presented here. The algorithm and results generated from this paper will be useful to all decision-makers who plan for disasters and emergencies, military or otherwise. Hospital administrators, payers, and government officials can use these methods to leverage real-world data to inform models of low-probability, high-risk events.

## Methods

### Study Design

**Figure 1.**
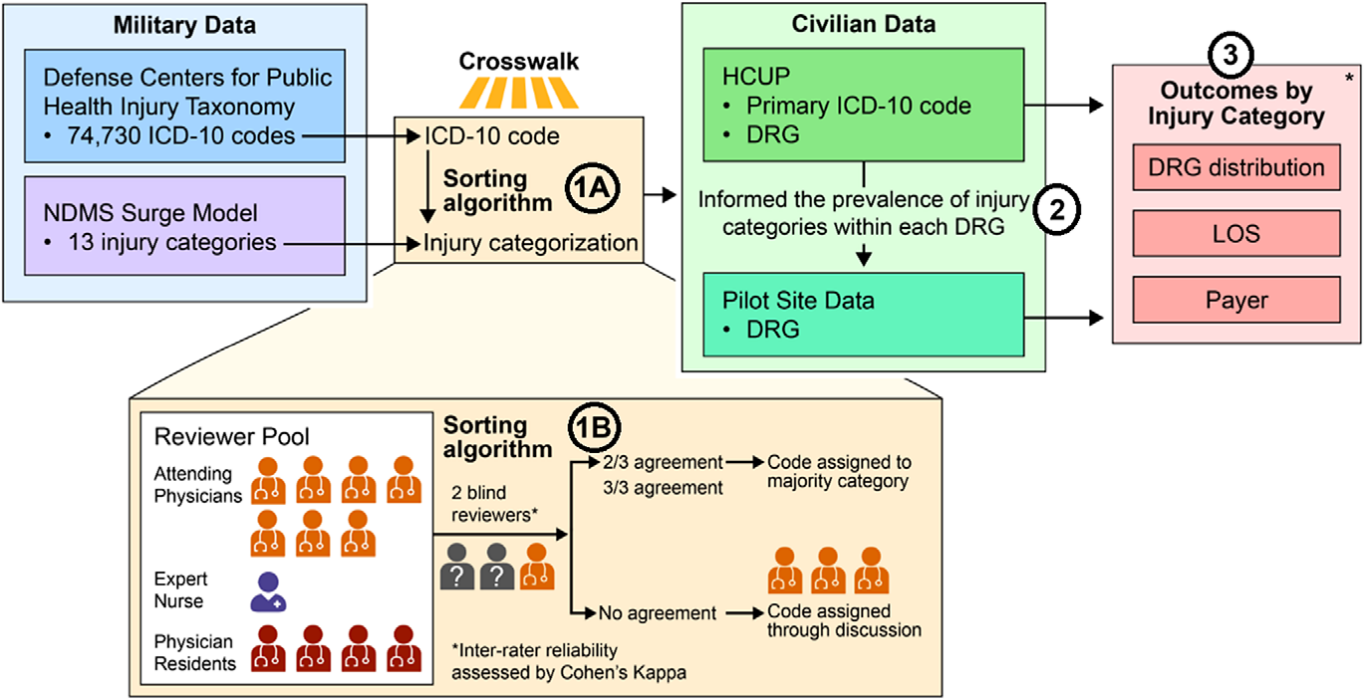
Methodology for linking broad predicted injury categories from the NDMS Surge Model (purple) to *International Classification of Diseases*, 10th Revision (ICD-10) codes (blue) using the customized code sorting algorithm (yellow box, Analysis Step 1). This allows historical civilian data from the Healthcare Utilization and Cost Project (HCUP, green) and civilian pilot site healthcare operations data (Pilot Site, teal) to be used to produce detailed descriptions of the DRGs, length of stay, and payer mix (pink) in each broad predicted injury category (Analysis Step 3). Colors are used to denote data sources in subsequent Tables. Numbers in circles correspond to Analysis Steps in the Methods section. *Note*: Since only DRGs are used in healthcare operations data, we compare calculated HCUP distributions with Pilot Site distributions, which are weighted by the prevalence of each DRG within a broad predicted injury category (Analysis Step 2).

This was an observational study with 2 stages. The first stage was development and validation of an algorithm that generated a crosswalk between broad predicted disaster injury categories and a military injury taxonomy of *International Classification of Diseases*, 10th Revision (ICD-10) codes. The second stage was application of this crosswalk to retrospective health care utilization data from the Healthcare Cost and Utilization (HCUP) project and Omaha-area health care systems, comprising NDMS Pilot Sites. The overall study design and analysis steps are summarized in Figure 1 and explained below.

### Data Sources

We used 4 data sources, which are highlighted in Figure 1: (A) A list of 16 broad disaster injury categories (grouped into 2 larger categories of “wounded in action” [WIA], or “disease and non-battle injuries” [DNBI]), as well as a predicted distribution across injury categories (called the “NDMS surge model,” purple in Figure 1), developed and validated by the NDMS team alongside other federal agencies, and physicians; (B) a taxonomy of all ICD-10 diagnosis codes, categorized into *military-relevant* injury and illness types, generated by the Injury Prevention Branch of the Defense Centers for Public Health^9^^,^^10^ (called the “Defense Centers for Public Health Injury Taxonomy,” blue in Figure 1); (C) 2019-2020 inpatient encounter data from the Healthcare Cost and Utilization Project (HCUP)^11^ for 3 states (Nebraska, Iowa, and Colorado), which contained both ICD-10 codes and DRGs, among other variables (called “HCUP,” green in Figure 1), accessed on July 31, 2024; (D) 2022-2023 hospital operations and financial data from 2 health systems (encompassing 5 hospitals) in the Omaha, Nebraska, USA, region and aggregated at the DRG-payer level (called “Pilot Site Data,” teal in Figure 1), accessed on November 5, 2024. Payers were categorized as commercial, Medicare, Medicaid, self-pay, and other. No identifying information about any individual was accessed in the study data sources.

Since a critical gap existed between the hospital data at the DRG level and the military data at the diagnosis code/injury category level, we developed the crosswalk as described below. To explore construct validity in an alternate, larger, more diverse health care setting, utilization and payer mix parameters were also calculated using inpatient data from HCUP for 3 states over the period 2019-2020.

#### Analysis Step 1A: Sorting algorithm

Several of the 16 broad predicted disaster injury categories from the “NDMS Surge model” (purple in Figure 1) were aggregated due to a lack of meaningful clinical difference in real-world civilian data between WIA and DNBI causes (e.g., fractures from either source were considered together) so that ultimately there were only 13 injury categories; these are summarized in Table 1. Next, a board-certified emergency physician assigned each ICD-10 code from the “Defense Centers for Public Health Injury Taxonomy” (blue in Figure 1) to 1 of the 13 broad predicted disaster injury categories from the “NDMS Surge model” (purple in Figure 1) by developing a customized ICD-10 code sorting algorithm. The sorting algorithm is provided in Supplementary Material 2. At the completion of this stage, each ICD-10 code was assigned to one (and only one) of the 13 broad predicted disaster injury categories.

**Table 1.** Broad predicted disaster injury categories (purple), predicted distribution of injured and ill servicemembers in a large-scale combat operation (LSCO), and corresponding *International Classification of Diseases*, 10th Revision (ICD-10) diagnosis codes (blue) matched via custom code-sorting algorithm

	Disaster Injury Category	Expected Proportion of All LSCO Patients	Unique ICD Codes (N)	Comments
Wounded in Action	Amputations	5.00%	194	
	Burns	3.80%	932	
	Fractures	19.6%	3,772	Aggregated with Musculoskeletal—Fracture
	Intracranial	1.60%	263	
	Nervous System	2.20%	346	
	Musculoskeletal	1.80%	3,152	Aggregated with Injury—Sprains and Strains
	Thoracic Open Wound	10.1%	237	
	Abdominal Open Wound	6.70%	421	
	Vascular Open Wound	10.5%	431	
	Other/Maxillofacial	5.70%	1,082	
Disease and Non-Battle Injury	Musculoskeletal—Fracture	6.10%	–	Aggregated with Fractures
	Injury—sprains and strains	7.80%	–	Aggregated with Musculoskeletal
	Digestive	3.60%	837	
	Mental disorder/Nervous system	3.90%	1,519	
	Other medical	9.40%	61,544	Aggregated
	Other surgical	2.20%		

*Note*: The number of unique ICD-codes indicates the number of ICD codes assigned to each broad predicted disaster injury category. Expected proportions were developed as part of the NDMS Surge Model, using existing literature^12^ and multiple stakeholder feedback from across the NDMS.

#### Analysis Step 1B: Validation and review

Once codes were thus assigned, 2 expert emergency clinicians reviewed approximately half of the ICD-10 codes included in all categories except “DNBI: Other Medical/Surgical.” The 2 expert emergency clinicians were drawn from a pool of 7 attending emergency physicians, 1 expert emergency nurse, and 4 senior emergency physician residents. The reviewer pool is described in yellow in Figure 1. All clinicians were blinded to the original physician’s crosswalk assignment. Reviewers were stratified by board-certified physician status, and each code was reviewed by at least 1 additional board-certified physician. Inter-rater reliability was assessed among all blinded reviewers using Cohen’s Kappa. Codes with 2 of 3 reviewers agreeing were assigned to the majority category. Codes with no agreement were assigned categories through discussion among board-certified physicians. In the case of poor agreement, additional validation of the remaining half of ICD-10 codes was planned.

#### Analysis Step 2: Application of crosswalk to real-world civilian utilization data

Next, we used HCUP inpatient data to match the ICD-broad predicted disaster injury category crosswalk to DRGs. We linked the crosswalk to the HCUP cohort using the ICD-10 code specified as the primary discharge diagnosis code for each HCUP encounter. Both the distribution of broad predicted disaster injury categories within each DRG and the distribution of DRGs within each broad predicted disaster injury category were calculated.

#### Analysis Step 3: Calculation of parameter distributions within real-world healthcare data

Once the crosswalk between ICD-10 codes, broad predicted disaster injury categories, and DRGs was complete, we could calculate the distributions of length of stay (LOS) and payer for each broad injury category separately using each of the HCUP and hospital cost-accounting datasets. In the HCUP data, which are encounter-level, we calculated the median and interquartile range (IQR) of LOS. In the health system “Pilot Site” data, which are aggregated at the DRG-payer level, we calculated a case-weighted median and IQR for LOS. In both datasets, we also calculated the distribution of payers (Medicare, Medicaid, commercial, self-pay, and other) for each broad predicted disaster injury category.

All analyses were conducted in R 4.2 (R Foundation for Statistical Computing). Cohen’s kappa was calculated using the *psych* package for R.

## Results

Table 1 shows the number of ICD-10 codes assigned to each broad injury category after the algorithm was applied, and expected patient distribution in the NDMS Surge Model. Overall, 74,730 ICD-10 codes were assigned to 1 of 13 categories. The “DNBI: Other Medical/Surgical illness” category comprised 61,546 ICD-codes, which were excluded from further cross-validation as, per the stakeholder-validated NDMS Surge Model described above, these are not expected to differ systematically between LSCO and usual civilian patient populations. Of the remaining 13,184 codes, 5,887 ICD-10 codes were cross-validated by at least 3 skilled clinicians. For 84.2% of the cross-validated ICD-10 codes, perfect agreement (3/3 blinded reviewers assigning the same category) was obtained; 99.2% of cross-validated codes had agreement between at least 2 raters. The Cohen’s Kappa statistic across all raters was 0.86, indicating nearly perfect agreement among the raters.^12^ Due to this agreement, the remainder were not validated.

Next, the crosswalk was applied to real-world civilian encounter data from 1,945,272 HCUP encounters from Nebraska, Iowa, and Colorado from 2019 and 2020. The mean, minimum, and maximum percent allocation of DRGs matching ICD-10 codes within each broad disaster injury category are summarized in Table 2. For example, in category “Burns,” some DRGs were assigned to this category every time they appeared in the HCUP encounter data (100% assignment), while other DRGs were never assigned to this category (0% assignment). On average, 0.78% of DRGs matched an ICD-10 code linked to “Burns” by the crosswalk. For most categories (except “WIA: Abdominal Open Wound,” “WIA: Amputations,” “WIA: Thoracic Open Wound, Fractures,” and “WIA: Nervous System,” and “DNBI: Mental Disorder / Nervous System”), at least one DRG was identified which was allocated to that broad predicted disaster injury category every time it appeared in the HCUP data (100% allocation).

**Table 2.** Mean percentage of each Diagnosis-Related Group (DRG) linked to each broad predicted disaster injury category (purple) in historical civilian data from the Healthcare Cost & Utilization Project (HCUP, green)

		Percentage of DRGs matching ICD-10 codes within disaster injury category		
	Disaster Injury Category	Mean % (SD)	Minimum	Maximum
Wounded in Action	Amputations	0.12 (1.91)	0.00%	48.3%
	Burns	0.78 (8.67)	0.00%	100%
	Fractures	3.93 (15.9)	0.00%	100%
	Intracranial	1.72 (11.3)	0.00%	100%
	Nervous System	0.38 (3.47)	0.00%	56.1%
	Musculoskeletal	1.98 (8.88)	0.00%	100%
	Thoracic Open Wound	0.37 (3.36)	0.00%	70.5%
	Abdominal Open Wound	0.69 (3.51)	0.00%	59.8%
	Vascular Open Wound	0.07 (0.63)	0.00%	10.6%
	Other/Maxillofacial	1.50 (7.51)	0.00%	77.6%
Disease and Non-Battle Injury	Digestive	10.4 (27.4)	0.00%	100%
	Mental Disorder/Nervous System	5.19 (19.3)	0.00%	100%
	Other Medical/Surgical	72.9 (37.6)	0.00%	100%

*Note*: For each civilian patient encounter, one disaster injury category was assigned using the ICD-10—Disaster Injury Category crosswalk described above. This table demonstrates, by disaster injury category, the mean (SD), minimum, and maximum proportion of each DRG which was linked to that disaster injury category.

The number of ICD-10 codes (in HCUP data), number of DRGs (determined by the crosswalk using HCUP data, as above), average LOS, and payer mix by broad predicted disaster injury category within the HCUP and Pilot Site hospital system data are presented in Table 3. Notably, median LOS ranged from 2.0 days (interquartile range, IQR 3.0) for both “WIA: Maxillofacial/Other and Musculoskeletal” categories in the pooled HCUP data, to 13.9 (IQR 15.8) for target health system care of WIA: Amputation category patients. Payer mix varied by disaster injury category (Table 3 and Supplementary Table 1).

**Table 3.** Encounters in civilian historical datasets, Healthcare Utilization Project (HCUP, green) and Pilot Sites (PS, teal), linked to each broad predicted disaster injury category (purple), demonstrating differences in length of stay for each broad predicted injury category. Differences in payer mix are illustrated using N (%) of encounters billing Medicare; full data for payers are in Supplementary Table 1.

	Broad Predicted Injury Category	Data source	Encounters (N)	ICDs (N)	DRGs (N)	LOS median (IQR)	Payer: Medicare, N (%)
Wounded in Action	Amputations	*HCUP*	351	92	22	3 (4.5)	56 (16.0)
		PS	1,751	–		13.9 (15.8)	683 (39.0)
	Burns	*HCUP*	1,905	247	23	5 (11.0)	444 (23.3)
		*PS*	1,720	–		7.8 (21.6)	841 (48.9)
	Fractures	*HCUP*	59,544	1,536	124	4 (4.0)	37,876 (63.6)
		*PS*	11,538	–		5.6 (6.6)	6,307 (54.7)
	Intracranial	*HCUP*	14,947	142	55	4 (6.0)	7,444 (49.8)
		*PS*	5,055	–		7.7 (11.1)	2,184 (43.2)
	Nervous System	*HCUP*	2,444	162	50	4 (10.0)	857 (35.1)
		PS	5,261	–		5.6 (8.9)	2,399 (45.6)
	Musculoskeletal	*HCUP*	18,544	881	123	2 (3.0)	9,830 (53.0)
		*PS*	9,211	–		5.0 (6.1)	4,913 (53.3)
	Thoracic Open Wound	*HCUP*	4,295	100	64	4 (5.0)	1,414 (32.9)
		*PS*	7,915	–		7.3 (6.4)	3,798 (47.9)
	Abdominal Open Wound	*HCUP*	3,067	247	124	4 (5.0)	616 (20.1)
		*PS*	10,342	–		5.8 (6.3)	4,667 (45.1)
	Vascular Open Wound	*HCUP*	*387*	103	18	3 (5.0)	46 (11.9)
		*PS*	1,867	–		12.9 (15.0)	715 (38.3)
	Maxillofacial/Other	*HCUP*	4,478	361	125	2 (3.0)	1,247 (27.8)
		*PS*	7,957	–		6.0 (8.2)	3,538 (44.5)
Disease and Non-Battle Injury	Digestive	*HCUP*	153,128	606	142	3 (3.0)	72,770 (47.5)
		*PS*	13,937	–		5.2 (4.9)	6,184 (44.4)
	Mental Disorder/Nervous System	*HCUP*	173,261	942	118	4 (4.0)	44,755 (25.9)
		*PS*	10,616	–		5.3 (6.8)	4,338 (40.9)
	Other Medical/surgical	*HCUP*	1,508,852	10,358	724	3 (3.0)	599,552 (39.7)
		*PS*	81,630	–		4.3 (4.9)	34,415 (42.2)

*Note*: ICD-10: *International Classification of Diseases*, 10th Revision. DRGs: Diagnosis-Related Groups. LOS: Length of stay.

## Limitations

As presented in Table 2, there were some broad predicted disaster injury categories in which few DRGs were mostly or completely allocated, namely, Amputations, Nervous System trauma, Thoracic and Abdominal Open Wounds, Vascular Open Wounds, and Other/Maxillofacial trauma. One hypothesis is that the broad predicted disaster injury categories which did not have a DRG 100% allocated to them are most likely to fall under the broad umbrella of multi-system severe trauma. To account for this, future analyses using the method developed here should use weighting to appropriately summarize patient needs arising from multi-system trauma.

Although the study’s use of real-world civilian health care utilization data to simulate the characteristics of military/disaster patients could be construed as a limitation, because it may be less applicable than military data itself, this was an intentional feature of the methods developed here, and contributes to their broad applicability in the prediction of patient needs during disasters. Further work could validate the patient characteristics against military or disaster data, if available.

## Discussion

This study presents a method to predict health care utilization characteristics of a predicted patient surge from retrospective civilian data. We developed and validated an algorithm that sorted ICD-10 diagnosis codes into a set of broad predicted disaster injury categories that are operationally relevant. The product of the algorithm was a crosswalk between ICD-10 codes, injury categories, and DRGs. We then applied the crosswalk to real-world civilian health care utilization data to predict disaster-patient parameters (e.g., frequency of patient-types, payers, and LOS).

Using this method can improve health care readiness for disasters, particularly across military-civilian collaborations such as NDMS, and enable accurate prediction of patient volume, hospital occupancy, staffing needs, and reimbursement during disasters. One of NDMS’s roles in disaster situations is to coordinate private and public health care systems to implement novel models of care. Given its key role in military-civilian cooperation during disasters, an NDMS activation is an important and unique application of this method.^13^^,^^14^

As a further example of the application of our novel method, we modeled changes in hospital occupancy and revenues during a theoretical overseas LSCO in which military patients comprising the 13 broad predicted injury categories used in the crosswalk were transferred back to the USA and cared for at 5 civilian hospitals in Omaha, Nebraska. The method we developed here was essential to link the broad predicted disaster injury categories to these hospitals’ own historical cost accounting data, which uses DRGs only. The linking allowed us to simulate occupancy and revenue outcomes by combining military injury projections and the real-world historical data from the 5 civilian hospitals, to ensure predictions were realistic. The results of this application are described in Weber et al.^15^ and can be used to inform reimbursement policy for civilian hospitals participating in the NDMS. For example, the expected proportion of military patients with burns according to the broad predicted injury categories (3.8%) was matched to 932 possible ICD-10 codes (see Analysis Step 1 and Table 1, above). HCUP data included 1905 encounters assigned to 247 of these ICD codes and 23 DRGs (see Analysis Step 2 and Tables 2 and 3). Real-world historical cost accounting data for the 5 civilian hospitals included 1720 encounters corresponding to these 23 DRGs. The encounters were weighted by the prevalence of each DRG in each broad predicted injury category (see Table 2) and then used to estimate the length of stay for these military patients with burns at the 5 civilian hospitals. This process resulted in an estimated length of stay of 7.8 (interquartile range, 21.6) days (see Analysis Step 3 and Table 3). Using these inputs, in the work by Weber et al.,^15^ we linked these predictions to the hospitals’ historical cost-accounting data, simulating the actual cost of caring for the predicted 3.8% of military patients with burns at these 5 civilian hospitals. This simulation allowed us to calculate the thresholds for hospital occupancy and reimbursement that would allow these 5 civilian hospitals to continue to operate while receiving military patients during an NDMS activation. Our methodology could be applied to other inputs to model both military and civilian disaster scenarios, informing disaster preparedness at the hospital, health system, state, or national levels.

Future work might apply similar techniques to obtain validated predictions about other specialized patient surges, for example, in the setting of viral outbreaks or large-scale domestic disasters. The broad predicted disaster injury categories used here were similar to those observed in a large-scale epidemiologic study of US military injuries during Operation Iraqi Freedom.^16^ However, one strength of the method developed here is its prospective nature and flexibility in using real-world clinical data to predict the needs of disaster patients, whether or not historical data for similar disasters are available. The use of multiple real-world health care data sources, such as HCUP and target hospital systems, and the broad agreement across the 2 different data sources, suggests that the method may be applicable to various types of health care data. Another strength is the near-perfect agreement obtained during blinded clinician validation of the ICD-10 crosswalk to broad predicted disaster injury categories.

An advantage of this method is the realistic nature of applying civilian data to transferred patients. Even if military treatment data were widely available, military treatment of patients may differ from civilian treatment, even under crisis standards of care. By aggregating less frequent conditions and injuries into broad predicted injury categories, it is possible to gain insights into how the civilian system might treat these types of injuries and conditions. This allows better prediction of how health care systems will fare when unexpectedly subjected to disasters comprised of low-frequency events. By applying this methodology, an individual hospital, health system, or regional coalition could improve simulations that model changes in volume, needed resources, as well as the financial impact of disaster scenarios.

The relative paucity of ICD-10 codes within each DRG, on average (as presented in Table 2), reflects the specialized nature of the injuries sustained in LSCO and reinforces the need for careful validation to predict the demands they would place on civilian hospital systems. This observation also underscores the importance of using clinically relevant categories to simulate patient needs in disasters, and suggests that DRGs alone may not adequately summarize the complexity of disaster patients, particularly those with injuries. Other literature has found that models relying on ICD-9 codes performed as well as specialized mortality prediction scores in trauma,^17^ lending support to their use in this method as well.

## Conclusions

Historic cases of patient surge and international to domestic transfers during LSCOs have highlighted the importance of health system readiness in responding to such events. As seen during the COVID-19 pandemic, health systems and public health officials use both broad predictions and disease-specific categories (such as ICD-10 codes) during disasters to understand health system stressors, but this can lead to barriers in addressing reimbursement concerns if they are not linked to health care operational data types such as DRGs. In addition, surge simulations often do not account for systematic differences in patient pathophysiology and care needs between surge and usual operations, which have significant impacts on capacity and reimbursement.^2^^,^^3^ The method presented in this paper links the way that health care and public health systems conceptualize disaster epidemiology with how health care systems conceptualize demand and reimbursement. Here, the method is validated specifically in the case of an LSCO with military and civilian patient transfer and NDMS activation. By adding real-world operational granularity to broad epidemiologic predictions about disasters, this method can be applied to predict population needs, health care system demand, and financial consequences during disasters, increasing health care readiness.

## Supporting information

10.1017/dmp.2026.10338.sm001 McCuskee et al. supplementary material 1 McCuskee et al. supplementary material

10.1017/dmp.2026.10338.sm002 McCuskee et al. supplementary material 2 McCuskee et al. supplementary material

## Supplementary material

The supplementary material for this article can be found at http://doi.org/10.1017/dmp.2026.10338.
