# Supplementary material for: Linking Disaster Predictions to Health Care Strain and Costs: A Novel Military-Civilian Case Study
Source: Disaster Med Public Health Prep. 2026 Mar 30;20:e65. doi: 10.1017/dmp.2026.10338 (PMC13107197; doi:10.1017/dmp.2026.10338)
Supplement: McCuskee et al. supplementary material 1 — McCuskee et al. supplementary material [file S1935789326103383sup001.docx]

*Supplementary Table 1: Encounters in civilian historical datasets, Healthcare Utilization Project (HCUP, green) and Pilot Sites (PS, teal), linked to each broad predicted disaster injury category (purple), demonstrating differences in payer mix.*

|  | *Broad Predicted Injury Category* | *Data source* | *Payer* | | | | |  |
| --- | --- | --- | --- | --- | --- | --- | --- | --- |
|  |  |  | Medicare, N (%) | Medicaid, N (%) | *Commercial, N (%)* | *Self-Pay, N (%)* | *Other, N (%)* | |
| **Wounded in Action** | Amputations | *HCUP* | *56 (16.0)* | *67 (19.1)* | *124 (35.3)* | *28 (8.0)* | *76 (21.7)* | |
|  |  | *PS* | *683 (39.0)* | *323 (18.5)* | *405 (23.1)* | *70 (4.0)* | *270 (15.4)* | |
|  | Burns | *HCUP* | *444 (23.3)* | *615 (32.3)* | *473 (24.8)* | *91 (4.8)* | *282 (14.8)* | |
|  |  | *PS* | *841 (48.9)* | *284 (16.5)* | *374 (21.74)* | *71 (4.1)* | *150 (8.7)* | |
|  | Fractures | *HCUP* | *37,876 (63.6)* | *5,363 (9.0)* | *12,388 (20.8)* | *1,203 (2.0)* | *2,714 (4.6)* | |
|  |  | *PS* | *6,307 (54.7)* | *1,365 (11.8)* | *2,341 (20.3)* | *356 (3.1)* | *1,169 (10.1)* | |
|  | Intracranial | *HCUP* | *7,444 (49.8)* | *2,312 (15.5)* | *3,932 (26.3)* | *463 (3.1)* | *796 (5.3)* | |
|  |  | *PS* | *2,184 (43.2)* | *791 (15.7)* | *1,283 (25.4)* | *210 (4.2)* | *587 (11.6)* | |
|  | Nervous System | *HCUP* | *857 (35.1)* | *385 (15.8)* | *948 (38.8)* | *59 (2.4)* | *195 (8.0)* | |
|  |  | *PS* | *2,399 (45.6)* | *777 (14.8)* | *1,334 (25.4)* | *156 (3.0)* | *595 (11.3)* | |
|  | Musculoskeletal | *HCUP* | *9,830 (53.0)* | *2,299 (12.4)* | *4,893 (26.4)* | *369 (2.0)* | *1,152 (6.2)* | |
|  |  | *PS* | *4,913 (53.3)* | *1,165 (12.65)* | *1,858 (20.2)* | *313 (3.4)* | *962 (10.4)* | |
|  | Thoracic Open Wound | *HCUP* | *1,414 (32.9)* | *849 (19.8)* | *1,563 (36.4)* | *218 (5.1)* | *251 (5.8)* | |
|  |  | *PS* | *3,798 (47.9)* | *1,080 (13.6)* | *1,958 (24.7)* | *284 (3.6)* | *795 (10.0)* | |
|  | Abdominal Open Wound | *HCUP* | *616 (20.1)* | *798 (26.0)* | *1,267 (41.3)* | *183 (6.0)* | *203 (6.6)* | |
|  |  | *PS* | *4,667 (45.1)* | *1,533 (14.8)* | *2,842 (27.5)* | *406 (3.9)* | *894 (8.6)* | |
|  | Vascular Open Wound | *HCUP* | *46 (11.9)* | *158 (40.8)* | *121 (36.4)* | *30 (7.8)* | *32 (8.3)* | |
|  |  | *PS* | *715 (38.3)* | *346 (18.5)* | *443 (23.7)* | *71 (3.8)* | *292 (15.6)* | |
|  | Maxillofacial/  Other | *HCUP* | *1,247 (27.8)* | *1,121 (25.0)* | *1,426 (31.8)* | *375 (8.4)* | *310 (6.9)* | |
|  |  | *PS* | *3,538 (44.5)* | *1,212 (15.2)* | *1,976 (24.8)* | *350 (4.4)* | *881 (11.1)* | |
| **Disease and  Non-Battle Injury** | Digestive | *HCUP* | *72,770 (47.5)* | *27,606 (18.0)* | *44,106 (28.8)* | *4,466 (2.9)* | *4,180 (2.7)* | |
|  |  | *PS* | *6,184 (44.4)* | *2,059 (14.8)* | *3,650 (26.2)* | *559 (4.0)* | *1,485 (10.7)* | |
|  | Mental Disorder / Nervous System | *HCUP* | *44,755 (25.9)* | *65,010 (37.6)* | *50,520 (29.2)* | *8,054 (4.7)* | *4,783 (2.8)* | |
|  |  | *PS* | *4,338 (40.9)* | *2,296 (21.6)* | *2,496 (23.51)* | *514 (4.8)* | *972 (9.2)* | |
|  | Other Medical/surgical | *HCUP* | *599,552 (39.7)* | *320,047 (21.2)* | *511,890 (33.4)* | *32,170 (2.1)* | *45,191 (3.0)* | |
|  |  | *PS* | *34,415 (42.2)* | *13,712 (16.8)* | *23,838 (29.2)* | *2,546 (3.1)* | *7,119 (8.7)* | |
